# 57 Nature based social prescribing for enhancing mental health and well-being

**DOI:** 10.1093/eurpub/ckae114.090

**Published:** 2024-09-26

**Authors:** Mary Lynch, Holly Whiteley, Ned Hartfiel, Rhiannon Tudor Edwards, Andrew Cuthbert

**Affiliations:** Royal College of Surgeons in Ireland, Dublin, Ireland; Bangor University, Bangor, Wales; Bangor University, Bangor, Wales; Bangor University, Bangor, Wales; Cardiff University, Cardiff, Wales

## Abstract

**Objectives:**

Nature Based Social Prescribing (NBSP) is a means of connecting people with non-clinical, nature-based community-led interventions delivered by 3rd sector organisations using local community assets. This research used a mixed-method Social Return on Investment (SROI) of a six-month pilot ‘Making Well’ NBSP programme to support individuals with chronic mental health conditions in Wales.

**Methods:**

The ‘Making Well’ programme costs were estimated by means of SROI along projected 20% overheads, were incorporated to reflect sustainable costs as the charity develops and more accurately estimate the future social value expected to be generated. The ‘Making Well’ project delivered two separate programmes between October 2021 and April 2022. Data was collected from participants (n = 12) at baseline and eight-week follow-up along with interviews to collect in-depth data on individuals lived experience of participating in the programme. The ‘Making Well’ programme costs were estimated and financial proxies from the HACT Social Value (SV) bank were applied to identified benefits.

**Results:**

The estimated cost of ‘Making Well’ programme inputs were £1,312 per participant and the net value of well-being benefits were £4,313 to £6,130 per participant, giving a range of SVR’s between £3.30 to £4.70 for every £1 invested in this NBSP intervention. The SROI forecast provides a three-year projection of the annual social value created and the estimated forecast cost of programme inputs was £797 per participant. The net value of well-being benefits was £4,313 to £6,130 per participant, giving a range of social value ratios between £5.40 to £7.70 for every £1 invested.

**Conclusions:**

The SROI results demonstrate that the ‘Making Well’ programme is an effective NBSP intervention for supporting people with enduring mild to moderate mental health conditions. This SROI contributes to emerging evidence on the use of community assets and NBSP in generating a return on investment and positive social value. SROI forecasting for socially prescribed interventions delivered by local community assets, such as The Fathom Trust, can help organisations demonstrate transparent and effective investment of public funds and support optimal future social value creation and long-term public health outcomes.

